# miR-486-3p mediates hepatocellular carcinoma sorafenib resistance by targeting FGFR4 and EGFR

**DOI:** 10.1038/s41419-020-2413-4

**Published:** 2020-04-20

**Authors:** Lin Ji, Zhongjie Lin, Zhe Wan, Shunjie Xia, Shi Jiang, Dong Cen, Liuxin Cai, Junjie Xu, Xiujun Cai

**Affiliations:** Key Laboratory of Laparoscopic Technology of Zhejiang Province, Department of General Surgery, Sir Run-Run Shaw Hospital, Zhejiang University School of Medicine, 310016 Hangzhou, China

**Keywords:** Liver cancer, miRNAs

## Abstract

HCC is a common malignancy worldwide and surgery or reginal treatments are deemed insufficient for advanced-stage disease. Sorafenib is an inhibitor of many kinases and was shown to benefit advanced HCC patients. However, resistance emerges soon after initial treatment, limiting the clinical benefit of sorafenib, and the mechanisms still remain elusive. Thus, this study aims to investigate the mechanisms of sorafenib resistance and to provide possible targets for combination therapies. Through miRNA sequencing, we found that miR-486-3p was downregulated in sorafenib resistant HCC cell lines. Cell viability experiments showed increased miR-486-3p expression could induce cell apoptosis while miR-486-3p knockdown by CRISPR-CAS9 technique could reduce cell apoptosis in sorafenib treatment. Clinical data also indicated that miR-486-3p level was downregulated in tumor tissue compared with adjacent normal tissue in HCC patients. Mechanism dissections showed that FGFR4 and EGFR were the targets of miR-486-3p, which was verified by luciferase reporter assay. Importantly, FGFR4 or EGFR selective inhibitor could enhance sorafenib efficacy in the resistant cells. Moreover, in vivo sorafenib resistant model identified that over-expressing miR-486-3p by lentivirus injection could overcome sorafenib resistance by significantly suppressing tumor growth in combination with the treatment of sorafenib. In conclusion, we found miR-486-3p was an important mediator regulating sorafenib resistance by targeting FGFR4 and EGFR, thus offering a potential target for HCC treatment.

## Introduction

Hepatocellular carcinoma (HCC) is a common malignancy worldwide, resulting in more than 700,000 deaths each year^1^. In China, HCC is the fourth most common malignancy and the third leading cause of cancer-related deaths due to the high prevalence of HBV infection. In 2012, approximately 50% of all new cases originated in China. Patients with BCLC stage 0 and 1 generally have better results after appropriate treatment, with 5-year survival rates ranging from 60 to 80%. However, many patients present with advanced HCC, for which surgery or regional treatment such as TACE is inadequate. In such cases, systemic therapy is required. However, unlike its neighbor intrahepatic cholangiocarcinoma, HCC rarely responds to chemotherapy. Thus, targeted therapy is likely to be a better option, but resistance is still a great issue. Among them, sorafenib, as the first targeted therapy for HCC, has been applied in clinical practice for more than 10 years and is especially a hot spot of drug resistance development. Sorafenib is an inhibitor of many kinases including VEGFR1, 2, and 3, PDGFRβ, c-Kit, and RET^2^, targeting both angiogenesis and tumorigenesis pathways. In two large clinical trials, sorafenib was demonstrated to extend survival time by 2–3 months in patients of both western and eastern groups^3,4^. Despite its efficacy, resistance emerges soon after initial treatment, but the mechanism is still unclear. Studies had focus on PI3K/AKT^5^, JAK/STAT pathways, hypoxia-inducible pathways^6^, and epithelial–mesenchymal transition^7^. Studies revealed that microRNAs (miRNAs) may have an important role in this resistance^8^ as well.

MiRNAs regulate a variety of biological processes post-transcriptionally. Target gene expression is usually downregulated when miRNA binds to its 3′UTR^9^. miRNAs are involved in cell proliferation, invasion, and many other biological behaviors of cancer, making them perfect biomarkers for cancer^10^. They are also reported to be good predictors for cancer prognosis. miR-21 is overexpressed in many cancers including HCC and is associated with a poor prognosis in patients^11^, while their roles in drug resistance are being increasingly reported. Studies show miRNAs regulate tamoxifen resistance in breast cancer^12^, cisplatin resistance in ovarian cancer^13^, and gemcitabine resistance in pancreatic ductal adenocarcinoma^14^ through various mechanisms. There have been several reports regarding the role of miRNAs in sorafenib resistance. MiRNAs confer resistance by directly regulating relevant mRNAs^15^ or cellular responses to treatment such as autophagy^16^. However, the mechanism is not fully understood.

In this study, we revealed that miR-486-3p is a pivotal sorafenib resistance mediator by regulating FGFR4 and EGFR, and thus reverse HCC sorafenib resistance in vitro and in vivo. This might provide potential therapeutic targets for novel combined therapies with sorafenib in HCC treatment.

## Results

### miR-486-3p expression is reduced in sorafenib-resistant cells and may participate in resistance development

**Fig. 1:**
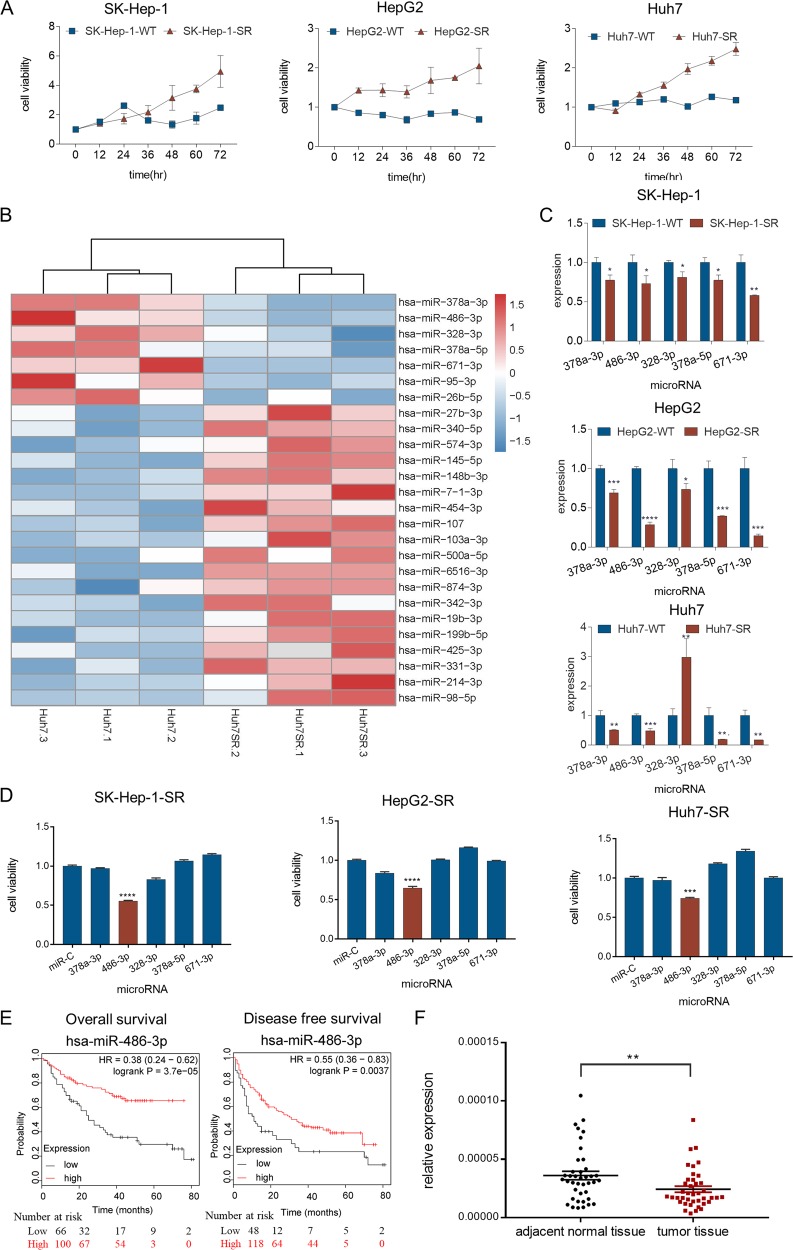
miR-486-3p expression is reduced in sorafenib-resistant cells and may participate in resistance development. **a** Cell viability measured by CCK-8 assay at different time points over 72 h showed that when cells were cultured in the same concentration of sorafenib (10 µM for SK-Hep-1 and Huh7, 7 µM for HepG2). The proliferation of resistant cells was greater than that of their parental cells; (**b**) miRNA sequencing of Huh7-SR and Huh7-WT showed a total of 26 miRNAs exhibited significantly different expression between the two groups. Five miRNAs, miR-671-3p, miR-378a-5p, miR-328-3p, miR-486-3p, and miR-378a-3p, were significantly reduced in Huh7-SR cells; (**c**) qRT-PCR evaluated the expression of the five candidates in all three resistant cell lines. The results confirmed that these five miRNAs were downregulated, with the exception of *miR-328-3p*, which was higher in Huh7-SR cells; (**d**) Cell viability measured by CCK-8 assay demonstrated *miR-486-3p* could consistently suppress resistant cell proliferation in all three resistant cells; (**e**) HCC prognosis data obtained from Kaplan Meier-plotter showed patients with higher *miR-486-3p* levels in cancer tissue had significantly better overall (HR = 0.38; 95%CI: 0.24 to 0.62*; P* = 3.7e−0.5) and disease-free survival (HR = 0.55; 95%CI: 0.36 to 0.83; *P* = 0.0037); (**f**) Clinical data with 40 pairs of HCC patients showed miR-486-3p levels was significantly lower in tumor tissue than adjacent normal tissue (*P* = 0.0044).

In an attempt to investigate the mechanism of sorafenib resistance in HCC, we introduced an in vitro model by culturing sorafenib-resistant cell lines with long-term exposure to sorafenib in the culture medium^7^. Resistance is considered to be achieved when cells can tolerate higher concentrations of sorafenib than the parental cell lines. Three resistant HCC cell lines were established: SK-Hep-1-SR, HepG2-SR, and Huh7-SR (Fig. 1a). Resistant cell lines were featured by higher cell viability in the presence of sorafenib than the parental cell lines.

The expression of many miRNAs has been associated with various processes in cancer. Evidences showed that miRNAs could be used as therapeutic agents has emerged^17^. Thus, we performed miRNA sequencing on Huh7-SR cells and its parental cell line, Huh7-WT, to identify potential miRNA candidates involved in the process of resistance development. The results indicated 26 miRNAs were different expressed between the two groups (Fig. 1b). Among them, five miRNAs, miR-671-3p, miR-378a-5p, miR-328-3p, miR-486-3p, and miR-378a-3p were significantly reduced in Huh7-SR cells. This reduced expression was also double-confirmed by qRT-PCR in SK-Hep-1-SR and HepG2-SR cell lines (Fig. 1c). To determine the biological functions of these miRNAs, we transfected the three resistant HCC cell lines with miRNA mimics and analyzed their responses to sorafenib, respectively. CCK8 assay showed that miR-486-3p mimics could consistently sensitize all three resistant cell lines to sorafenib (Fig. 1d).

To further determine the value of miR-486-3p in the clinical practice, we referred to Kaplan Meier-plotter online database^18^. Analyses showed that in the liver cancer database, patients with significantly higher levels of miR-486-3p had better overall survival (HR *=* 0.38; 95%CI: 0.24 to 0.62; *P* *=* 3.7 × 10^−5^) and better disease-free survival (HR *=* 0.55; 95%CI: 0.36 to 0.83; *P* *=* 0.0037) (Fig. 1e). These results indicated that the low level of hsa-miR-486-3p was probably relevant to a poorer treatment response. We further tested miR-486-3p levels in the samples of 40 patients, and found that miR-486-3p levels was downregulated in tumor tissue than adjacent normal tissue (*P* *=* 0.0044) (Fig. 1f), indicating miR-486-3p was also important in tumorigeneses.

Through miRNA sequencing of sorafenib-resistant cells, we discovered that miR-486-3p was downregulated in sorafenib resistant cells. In addition, in vitro experiments and clinical data both indicated that miR-486-3p played a role in drug resistance development.

### miR-486-3p could sensitize cellular response to sorafenib

**Fig. 2:**
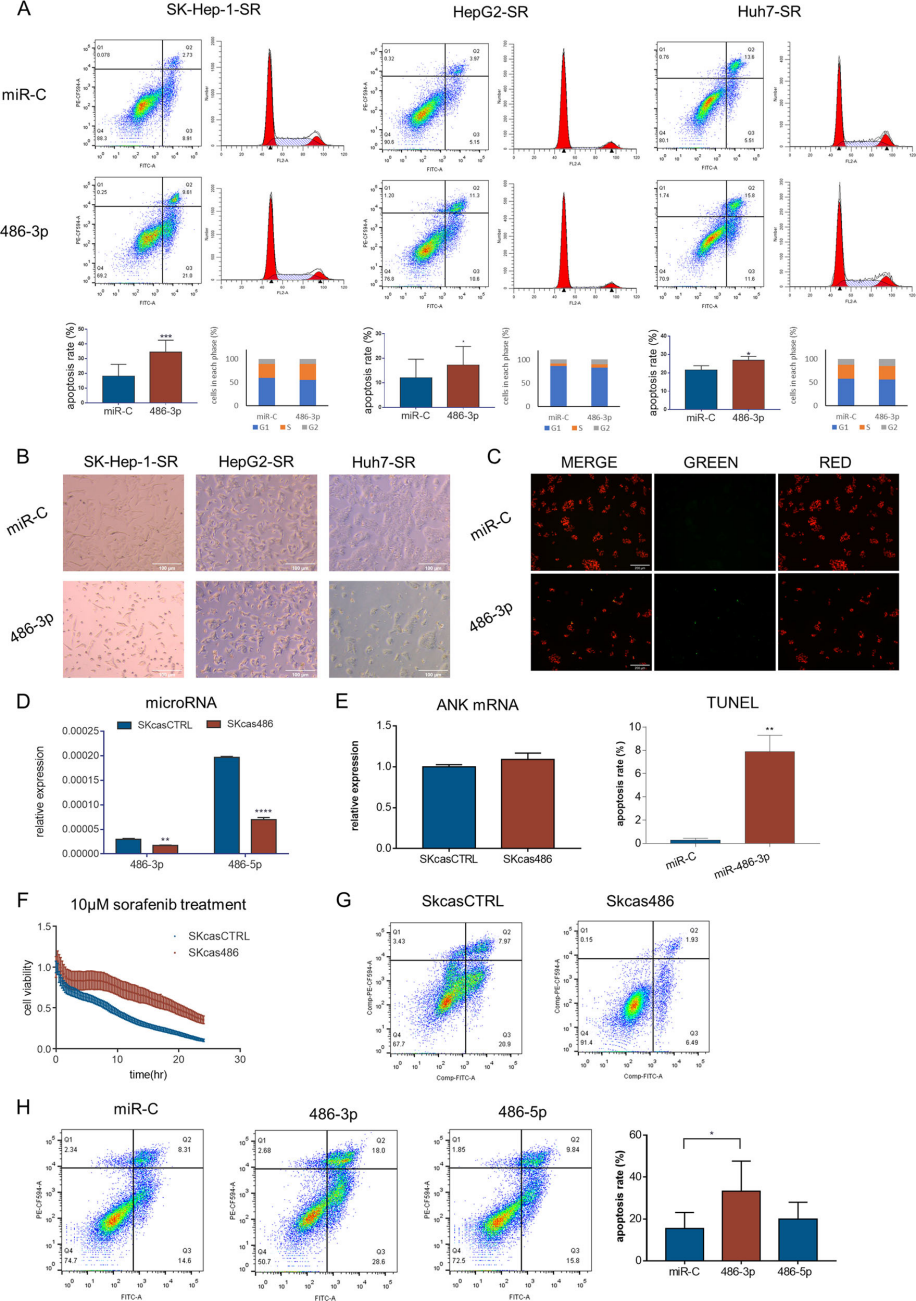
miR-486-3p could sensitize cellular response to sorafenib. **a** Flow cytometry showed miR-486-3p transfection could induce apoptosis in resistant cells but did not affect cell-cycle distribution; (**b**) Microscopy image post-miR-486-3p transfection and incubation with sorafenib for 48 h. miR-486-3p induced significant cell death; (**c**) TUNEL assay showing miR-486-3p induced apoptosis in HepG2-SR cells; (**d**) qRT-PCR showing miR-486-3p and miR-486-5p levels were downregulated in SKcas486 cells compared with SKcasCTRL; (**e**) qRT-PCR showing the host gene of miR-486-3p and miR-486-5p was not changed in SKcas486 cells; (**f**) Real-time cellular analysis showed SKcas486 was more tolerant to sorafenib treatment; (**g**) Flow cytometry using PI/Annexin V showed sorafenib induced less apoptosis in SKcas486 cells compared with SKcasCTRL; (**h**) Transfection with miR-486-3p in SKcas486 cells caused significant cell apoptosis whereas miR-486-5p did not.

Previous results showed miR-486-3p could suppress cell proliferation, thus we sought to investigate the mechanisms by which this occurred. Through in vitro tests, we found that increasing miR-486-3p levels by transfection of mimics could induce cell apoptosis, as observed by FACS analyses, cell morphological changes and TUNEL assays (Fig. 2a–c). However, cell cycle distribution did not change significantly after transfection (Fig. 2a). These data confirmed that miR-486-3p could sensitize resistant cells to sorafenib by inducing apoptosis.

To further verify our hypothesis, we employed CRISPR-CAS9 technique to knock down hsa-miR-486-3p in SK-Hep-1 cells. We successfully generated SK-Hep-1-cas486 cells with lower levels of both hsa-miR-486-3p and hsa-miR-486-5p (Fig. 2d). Since there was no clear border of primary miRNA, we extended the precursor miRNA by 200 bp at both ends and used this sequence as a template. Then, we constructed a miR-486-3p knockdown cell line in accordance with previous reports^19,20^. Because the process of miRNA splicing is quite different from mRNA, all the details of this process are not yet clear. But in our system, both miR-486-3p and miR-486-5p levels examined by qRT-PCR were significantly reduced in SKcas486 cells while their host gene ANK1 did not show a significant change (Fig. 2e). Using real-time cellular analysis, we found SKcas486 cells were more resistant to sorafenib than SKcasCTRL cells (Fig. 2f). At the same time, flow cytometry also showed sorafenib induced less apoptosis in SKcas486 cells (Fig. 2g). Since both miR-486-3p and miR-486-5p levels were decreased in SKcas486 cells, it was still unclear as to which miRNA was involved in the process. To further differentiate the roles of miR-486-3p and miR-486-5p in response to sorafenib, we increased miR-486-3p and miR-486-5p levels in SKcas486, respectively. We then found miR-486-3p could induce significant apoptosis whereas miR-486-5p could not (Fig. 2h). Therefore, we believed the resistance of SKcas486 to sorafenib was induced by decreasing miR-486-3p.

In summary, we found that miR-486-3p could induce apoptosis in resistant cells while decreasing its expression could enhance cellular tolerance to sorafenib.

### miR-486-3p participated in sorafenib resistance by targeting FGFR4 and EGFR

**Fig. 3:**
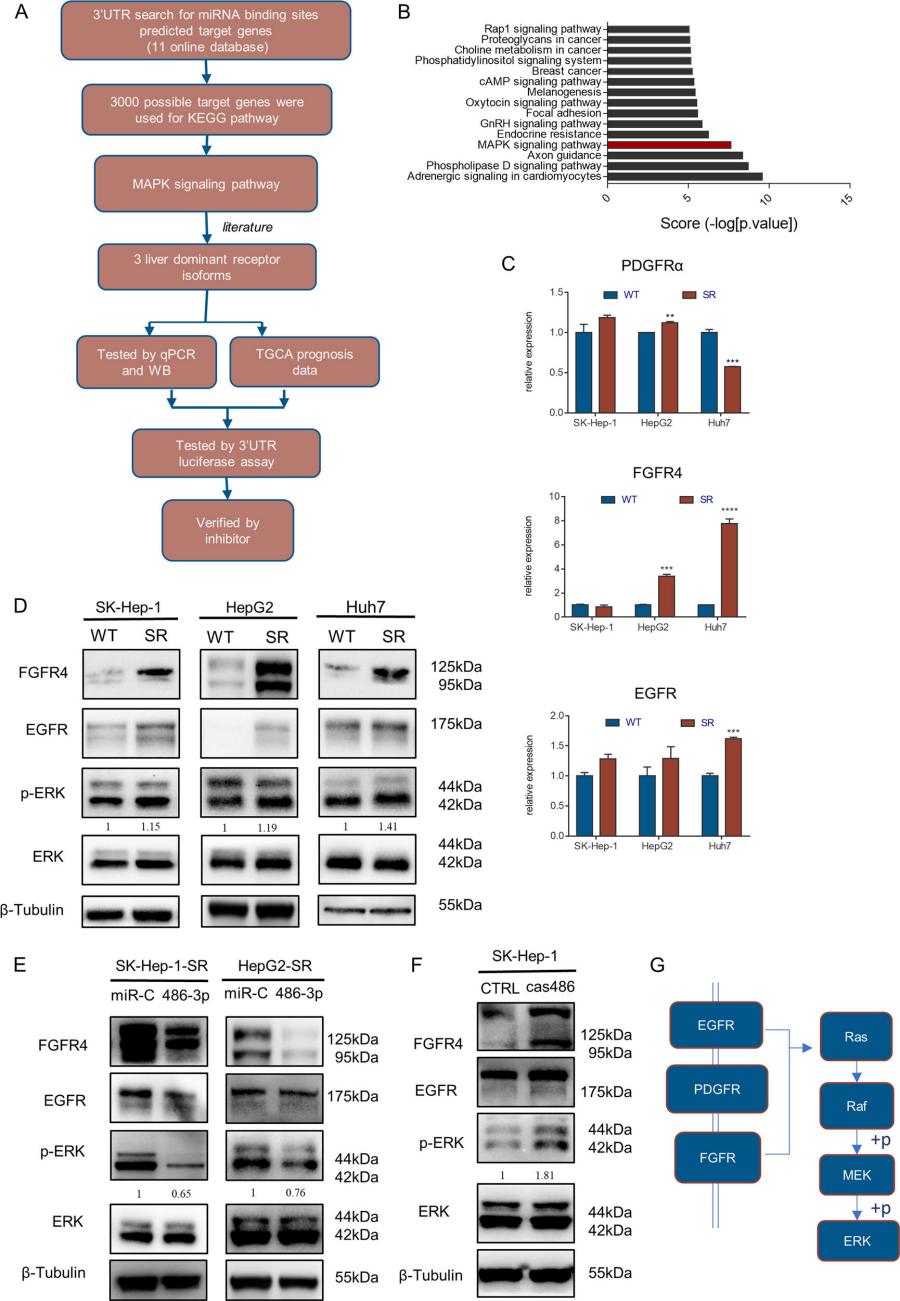
miR-486-3p participated in sorafenib resistance most likely by targeting FGFR4 and EGFR. **a** Schematic representation of the workflow to miR-486-3p-target genes; (**b**) Three thousand possible target genes were included for KEGG pathway analysis. Top fifteen overrepresented processes were sorted by score (−log [*p* value]). A highly positive score suggested this pathway had many possible targeted sites and did not have much sites untargeted. Results indicated MAPK signaling pathways were most likely to be targeted by miR-486-3p; (**c**) qRT-PCR revealed mRNA levels of FGFR4 were significantly higher in HepG2-SR and Huh7-SR cells compared with their parental lines. mRNA levels of EGFR were significantly higher in Huh7-SR cells. mRNA levels of PDGFRA were significantly lower in Huh7-SR; (**d**) WB showed FGFR4, EGFR were significantly upregulated in resistant cell lines along with their common downstream target pERK; (**e**) WB demonstrated miR-486-3p transfection reduced FGFR4 and EGFR levels; (**f**) SKcas486 cells had higher levels of these proteins. Changes in protein levels were consistent with pERK, the downstream protein; (**g**) A potential model of miR-486-3p targets.

In our study, we demonstrated that miR-486-3p participated in sorafenib resistance. To study the detailed molecular mechanisms, we used MiRWALK2. 0 to predict potential targets of miR-486-3p^21^. A total of 12 databases were used in this procedure (Fig. 3a). Candidates were sorted according to the number of databases in which they were predicted, and the top 3000 possible targets of miR-486-3p were included for KEGG pathway analysis (Fig. 3b). Results showed fifteen pathways were most likely to be targeted. We then focused on MAPK pathway as it was also highly involved in HCC development and sorafenib resistance. Sorafenib functions as a kinase inhibitor. We noticed many tyrosine kinase receptors in this pathway had been predicted. Three particular tyrosine kinase receptors from those candidates, namely FGFR4, EGFR and PDGFRα (Fig. 3g)^22,23^ were reported to be highly associated with HCC occurrence and development but are not direct targets of sorafenib^24^. We then measured mRNA levels of those receptors in resistant and parental cell lines by qRT-PCR, showing FGFR4 mRNA levels were significantly higher in HepG2-SR and Huh7-SR cells, EGFR mRNA levels were significantly higher in Huh7-SR, PDGFRA mRNA levels were distinctly lower in Huh7-SR (Fig. 3c). Then, we measured FGFR4 and EGFR protein levels. Results showed the protein levels of FGFR4 and EGFR were significantly higher in sorafenib resistant cells. Consistently, pERK levels were also higher in resistant cells (Fig. 3d). WB also revealed that transfection of miR-486-3p mimics could suppress the expression of these proteins along with their downstream target pERK (Fig. 3e). Furthermore, FGFR4 and EGFR levels in SKcas486 were higher than in SKcasCTRL, along with pERK levels (Fig. 3f). To further prove our hypothesis, we conducted an RNA-seq using Huh7-SR cells with or without miR-486-3p mimics transfection. Results indicated various altered genes including those closely related with FGFR4 and EGFR (Supplementary Figs. 1, 2).

In this part, we found miR-486-3p could contribute to sorafenib resistance mainly through targeting FGFR4 and EGFR.

### miR-486-3p suppressed the protein expression of FGFR4 and EGFR by targeting their 3′UTRs

**Fig. 4:**
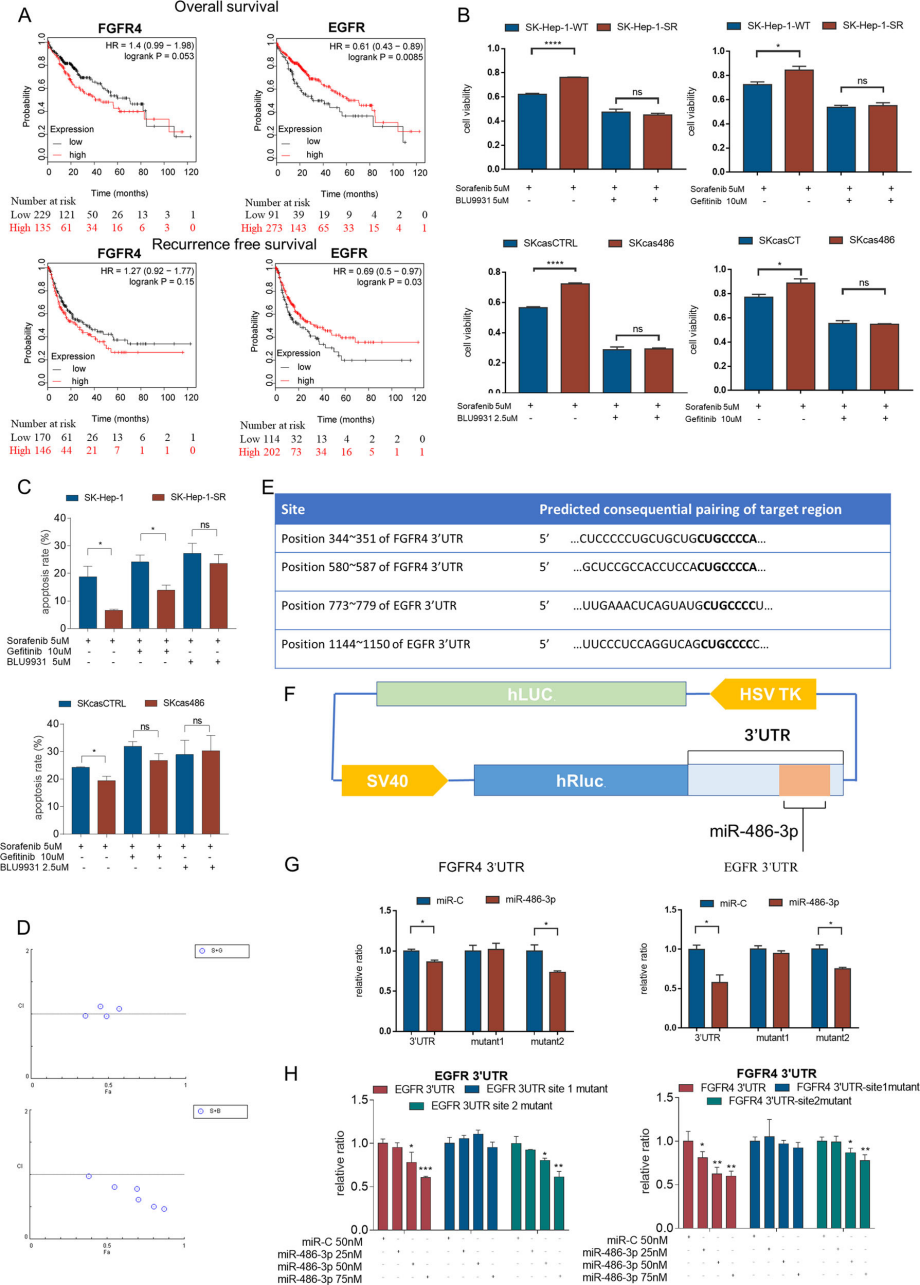
miR-486-3p suppressed proliferation of resistant cells by targeting the 3′UTR of FGFR4 and EGFR. **a** Kaplan Meier-Plotter analysis showed FGFR4 levels were negatively related to HCC patient prognosis. However, patients expressing higher levels of EGFR had better clinical results; (**b**) FGFR4 or EGFR inhibitor could sensitize SK-Hep-1-SR and SKcas486 cells to sorafenib treatment; (**c**) apoptosis assay showed FGFR4 or EGFR inhibitor could induce apoptosis in SK-Hep-1-SR and SKcas486 cells under sorafenib treatment. **d** CI analysis were performed using ComboSyn. Results indicated that sorafenib and Gefitinib exerted weak synergistic effect in vitro experiments. Sorafenib and BLU9931 had strong synergistic effect. **e** According to the online database TargetScan, there were two predicted targets for the 3′UTR of FGFR4 or EGFR; (**f**) Schematic structure of luciferase reporter vector containing both firefly and Renilla luciferases. Wild-type or mutant 3′UTR sequence of FGFR4 or EGFR located after Renilla luciferase; (**g**) Dual-luciferase reporter assay showed miR-486-3p reduced expression of Renilla luciferase with wild-type FGFR4 3′UTR or a mutated second site but not a mutated first site. Result was similar with EGFR 3′UTR; (**h**) Dual-luciferase reporter assay showed increasing miR-486-3p dose could enhance inhibition effect on Renilla luciferase.

Because the mRNA levels of PDGFRA were quite disaccorded with miR-486-3p level in cell lines, we postulated that miR-486-3p may impact cell apoptosis by targeting FGFR4 or EGFR. Then, we examined the effects of the candidate targets on HCC prognosis using an online database Kaplan Meier-plotter^18^, which showed that high levels of FGFR4 may be related to poorer overall survival (*P* = 0.053) and recurrence free survival (*P* = 0.15)^25^, while, EGFR had adverse prognostic data (Fig. 4a). These results indicated that the mRNA levels of FGFR4 and EGFR might not be ideally correlated with HCC prognosis, possibly due to their post-transcriptional regulations. Moreover, many studies reported EGFR took part in sorafenib resistance^23^. Taken all these experimental results into consideration, we assumed miR-486-3p may exert its effect through targeting FGFR4 and EGFR.

To further confirm our hypothesis, an FGFR4-specific inhibitor, BLU9931, and an EGFR-specific inhibitor, gefitinib, were used to treat SK-Hep-1-SR and SKcas486 cells combined with sorafenib, comparing to the parental cells and control cells. Interestingly, BLU9931 and gefitinib could reverse sorafenib resistance in SK-Hep-1-SR or SKcas486 cells (Fig. 4b). This was verified by apoptosis assay (Fig. 4c) Combination index (CI) analysis was performed using COMPUSYN as reported previously^26^. Result showed gefitinib and sorafenib had weak synergistic effects. While, sorafenib and BLU9931 had strong synergistic effects. (Fig. 4d)

There were two potential targets of miR-486-3p in the 3′UTR of FGFR4 and EGFR according to TargetScan (Fig. 4e)., Dual-luciferase reporter assays verified the suppressive effects of miR-486-3p on the translational level of FGFR4 and EGFR (Fig. 4f, g). We then mutated these sites one by one to determine which site was more important. We found that miR-486-3p mainly targeted the first site in 3′UTR of FGFR4 and EGFR (Fig. 4f, g) indicating that it exerted its function mainly through the first site. Luciferase reporter assay was also performed using different dose of miR-486-3p. Result showed increasing microRNA level could increase suppressive effects on the translational level of FGFR4 and EGFR (Fig. 4h)

Thus, all in vitro experiments, online datasets, and clinical data in this study revealed that miR-486-3p could induce apoptosis of sorafenib resistant cell through targeting FGFR4 and EGFR, which was mediated by binding to specific sites the their 3′UTR.

### miR-486-3p could suppress cell proliferation and contribute to sorafenib resistance in vivo

**Fig. 5:**
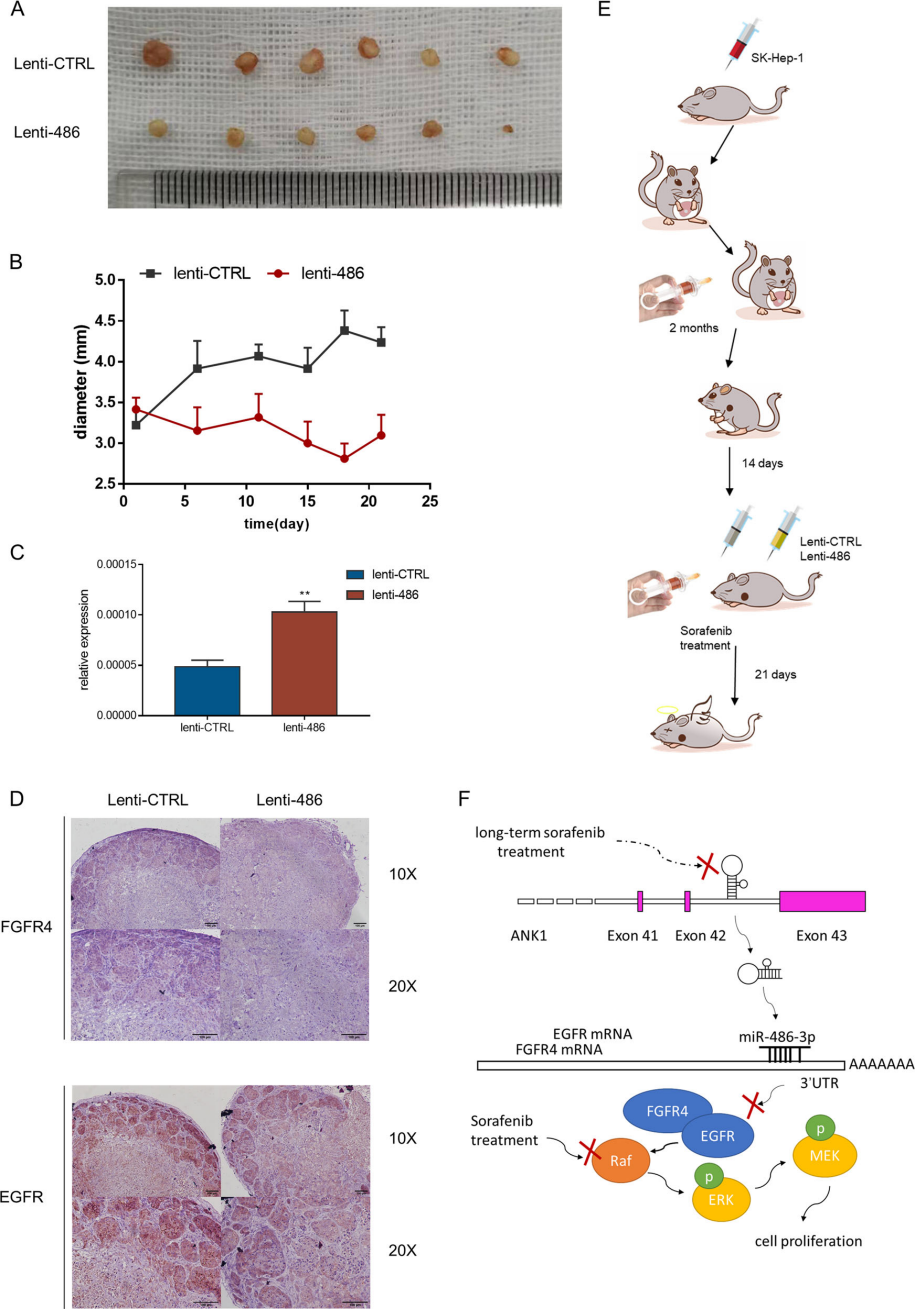
in vivo experiment showed miR-486-3p overexpression could enhance sorafenib effect. **a** Gross view of tumors from both groups; (**b**) Tumor size was measured every 3–5 days after lentivirus interference; (**c**) miRNA levels of two groups were verified using qRT-PCR; (**d**) Immunohistochemistry showed increased miR-486-3p level could suppress FGFR4 and EGFR level in vivo*;* (**e**) Schematic representation of the in vivo model timeline. A total of 6 mice were included in each group; (**f**) Functional model of the tumor suppressor miR-486-3p.

An in vivo sorafenib resistant model was introduced to investigate the role of miR-486-3p and to explore the possibility of using this miRNA as a treatment method in clinic settings (Fig. 5e). Orthotopic SK-Hep-1 cell derived xenograft (CDX) HCC model was established as described in previous studies^27–29^. After tumor formation, tumor was harvested, cut into small pieces and transferred to the livers in 4-week-old BALB/C nude mice. Those mice carrying tumors were administrated with 30 mg/kg/d sorafenib for another two months. After two-month sorafenib treatment, tumors were regarded to be sorafenib resistant. Subcutaneous tumor models were constructed according to a previous report^30^. Pieces from one sorafenib resistant tumor were implanted to axillary areas in 4-week-old BALB/C nude mice. Fourteen days later, a total of twelve mice bearing similar size of resistant tumors were included and allocated to two groups. Treatment using lentivirus was conducted by peritumoral injection of lentivirus overexpressing miR-486-3p or mock (10^7^ units in 50 μl PBS). In the meantime, mice of both groups were administrated with sorafenib treatment. Tumor status was measured every 3–5 days. After another three weeks, mice were sacrificed. We found increasing miR-486-3p could significantly enhance sorafenib efficacy on in vivo sorafenib resistant tumors (Fig. 5a, b). The levels of miR-486-3p in two groups were verified by qPCR (Fig. 5c). In addition, immunohistochemistry was performed to evaluate the protein levels of FGFR4 and EGFR in these treated tumors (Fig. 5d). Results showed miR-486-3p could reduce the protein levels of FGFR4 and EGFR in vivo. Thus, we revealed that miR-486-3p could be a promising novel combined therapy to help overcome sorafenib resistance in HCC patients (Fig. 5f).

**Fig. 6:**
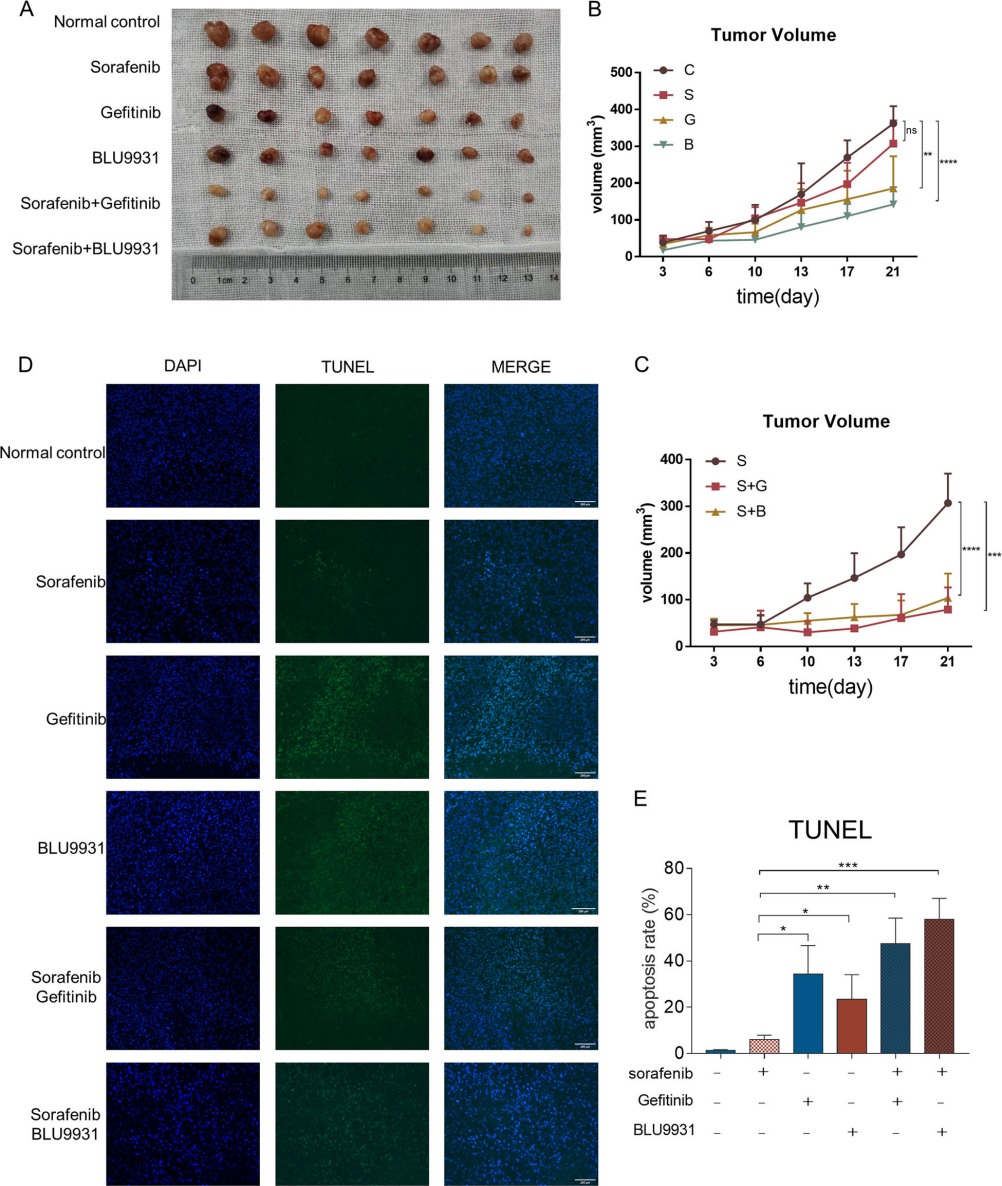
in vivo experiment showed Gefitinib and BLU9931 could sensitize resistant tumor to sorafenib treatment. **a** Gross view of tumors from 6 groups. **b** There was no significant difference between sorafenib treatment and control group (*p* = 0.0575). Gefitinib (*p* = 0.0038) and BLU9931 (*p* < 0.0001) could reduce sorafenib resistant tumor size. **c** Result showed Gefitinib (*p* < 0.0001) and BLU9931 (*p* < 0.0001) could enhance sorafenib effect. **d** TUNEL assay showed Gefitinib and BLU9931 could induced apoptosis in sorafenib resistant tumors. **e** Quantified result of TUNEL assay.

We also used the in vivo sorafenib resistant model to explore the combination effect between sorafenib, gefitinib and BLU9931 (Fig. 6a). A total of 42 mice were used in this experiment. Sorafenib resistant mouse model was established as previously described. Treatment was initialed when tumors reached 2 mm in diameter. Mice were separated into 6 groups randomly. Each group included 7 mice. Mice were treated with vehicle solution, sorafenib 30 mg/kg/d, gefitinib 150 mg/kg/d, BLU9931 50 mg/kg, twice daily, the combination of sorafenib and gefitinib, or the combination of sorafenib and BLU9931. All treatments were administrated orally. Size of tumor was measured every 3–4 days. After 3 weeks, mice were sacrificed and tumors were collected for further investigation. Two-way ANOVA analyses were used. 2 independent experiments were performed.

Results showed that we successively established sorafenib resistant mouse model with no significant difference between control group and sorafenib treatment group (*p* = 0.0575). Gefitinib (*p* = 0.0038) and BLU9931 (*p* < 0.0001) alone could reduce tumor volume in in vivo sorafenib resistant model (Fig. 6b). Gefitinib (*p* < 0.0001) and BLU9931 (*p* < 0.0001) could enhance sorafenib effect in sorafenib resistant tumors. (Fig. 6c)

TUNEL assay was conducted to investigate the apoptosis level in these tumors. Results showed Gefitinib and BLU9931 could induce apoptosis in sorafenib resistant tumors (Fig. 6d, e). This indicated EGFR and FGFR4 played a critical role in resistance development.

## Discussion

HCC is a worldwide health problem. It is the fourth most common cause of cancer-related death, with a 5-year survival rate of 18%. Pathways including Notch, PI3K/AKT, ERK, mTOR, MAPK, Hedgehog, and Wnt are all regarded to be important in the development and progression of HCC^31^. A comprehensive understanding of those pathways helps us to develop more effective treatments for HCC. As mentioned before, sorafenib is an inhibitor of many kinases involved in the MAPK signaling pathway. It has benefited many advanced HCC patients in clinic, but drug resistance still remains a major pitfall. Thus, combined therapies based on sorafenib resistance mechanisms are in urgent need.

Some growth factor signaling pathways have emerged as critical players in the process, especially FGF^32,33^. Indeedm studies found that FGFR played a vital role in drug resistance^34^. Lenvatinib, a multiple kinase inhibitor targeting FGFR showed optimistic prospect in treating HCC^35^. There are four isoforms of FGFR, which encode the tyrosine kinase receptors, while FGFR4 is the predominant isoform in human hepatocytes^36^. Activation of FGFR4 can phosphorylate FGF receptor substrate 2, recruit growth factor receptor-bound protein 2, and then activate the RAS/RAF/MEK/ERK and PI3K-AKT pathways^37^. Evidence show FGFR4 and its specific ligand FGF19 are highly expressed in primary HCC^38,39^. Furthermore, overexpression of FGF19 could induce HCC in a mouse model^40^, FGF19 was proven to induced hepatocyte proliferation through FGFR4 activation^41^. Evidence also shows downregulation of the FGF19/FGFR4 pathway could lead to decreased viability, invasion, and tumor formation of HCC in SCID mice^42^. This all indicates a crucial role for FGF19/FGFR4 in HCC. A previous study also demonstrated that the FGF19/FGFR4 pathway is involved in the acquisition of sorafenib resistance. The results showed that when overexpressed, FGF19 could reduce apoptosis by inhibiting the effect of sorafenib on ROS generation. Furthermore, loss of FGF19 or its receptor FGFR4 could help enhance ROS generation by sorafenib^34,43^. These previous studies demonstrate that molecules targeting FGFR4 may sensitize HCC cells to sorafenib treatment.

EGFR has been demonstrated to be a driver of tumorigenesis especially in lung, breast cancer and glioblastoma^44^. Its overexpression in HCC had long been recognized^45^. EGFR blockade was then proved to be a potential target in treating HCC. Recently, researchers also found its role in sorafenib response. EGFR activation is a potential determinant of primary sorafenib resistance^23^. Meanwhile, the activation of EGFR pathway also contributes to acquired resistance as a result of HIF-2α upregulation induced by sorafenib^46^. In addition, a combination of EGFR inhibitors and sorafenib results in better control over HCC. These previous studies reveal that EGFR inhibitor may help overcome sorafenib resistance.

In this study, we found hsa-miR-486-3p could regulate sorafenib response in HCC by targeting both FGFR4 and EGFR, making it a better therapeutic target than FGFR4 and EGFR inhibitors. Through microRNA sequencing, we found that hsa-miR-486-3p was downregulated in sorafenib-resistant cells. Hsa-miR486-3p is reported in the literature to regulate BCL11A or MAF expression in human erythroid cells^47,48^. It is also recognized as a stable marker in the acute coronary syndrome^49^, and is associated with metastasis in cervical cancer patients by targeting ECM1^50^. However, its role in HCC has not yet been reported. An online database indicated its expression as positively correlated with OS and DFS of HCC patients. Our clinical results also indicated that miR-486-3p levels was downregulated in tumor tissue than adjacent normal tissue. Subsequent in vitro and in vivo experiments confirmed downregulating of miR-486-3p could contribute to sorafenib resistance, and increasing its level could re-sensitize HCC cells to sorafenib therapy.

Recently, oligonucleotide therapeutics have emerged as a potential treatment method. Some have entered clinical trials to test their validity. MiR-29 is reduced in various fibrotic conditions^51^, and subcutaneous miR-29 supplementation is currently being assessed for its role in scarring in two clinical trials (NCT02603224 and NCT03601052). MiR-122 is essential to the propagation of HCV RNA. The use of miravirsen of miR-122 exhibits therapeutic effect to downregulating HCV RNA levels^52^. Here in our study, in vivo sorafenib resistant model experiments showed that subcutaneous injection of lentivirus overexpressing miR-486-3p could enhance sorafenib efficacy, offering a possible target to overcome sorafenib resistance in HCC patients.

However, there are still some inadequacies in this study. Since sorafenib is mainly administrated in patients with advanced HCC who usually lost surgery opportunity, it is difficult to obtain sample tissues from those patients. Although in vivo sorafenib resistant animal model demonstrated the specific role of miR-486-3p, the impact of miR-486-3p in sorafenib tolerances of HCC patients need further investigation.

In conclusion, we found that miR-486-3p is a critical sorafenib resistance mediator by regulating FGFR4 and EGFR, and thus providing a potential target for HCC treatment.

## Materials and methods

### Cell culture

3 human HCC cell lines (SK-HEP-1, HepG2, and Huh7) used in this study, were all purchased from the American Type Culture Collection (ATCC, Manassas, VA, USA). Cell culture was according to the manufacturer’s protocol and all the cell lines were grown in DMEM supplied with 10% FBS at 37 °C with 5% CO2.

### Oligonucleotide transfection

miRNA mimics (miR-486-3p, miR-486-5p, miR-671-3p, miR-378a-5p, miR-328-3p, miR-378a-3p) and negative control miRNA were all purchased from Ribobio (Guangzhou, China) and GenePharma (Shanghai, China). miRNA mimics and negative control were transient transfected into HCC cells using Lipofectamine 3000 reagent (Invitrogen, USA) or Lipofectamine RNAiMAX Reagent (Invitrogen, USA) with a working concentration of 50 nM according to the manufacturer’s protocol. miRNA mimics effect was confirmed by qRT-PCR at 48 h post transfection.

### Cell viability test

Transiently transfected cells were seeded on a 96-well (0.5–1 × 10^4^/well) or 24-well (1–2 × 10^4^/well) plate with 3 replicates. Then, cells were incubated with sorafenib for 48~72 h. After incubation, cell viability was assessed according to the Cell Counting Kit-8 (CCK-8) kit (Yeasen, Shanghai, China).

SKcas486 and SKcasCTRL cells were seeded on a 96-well (0.5 × 10^4^/well) E-Plate (ACEA Biosciences, Hangzhou, China) according to the manufacture’s protocol. Culture medium was supplied with different concentration of sorafenib after cell adherence. Incubation lasted for more than 72 h and data was collected by xCElligence RTCA MP (ACEA Biosciences, Hangzhou, China).

### Apoptosis assay

Cell apoptosis was determined with an PI/annexin V-FITC apoptosis kit (MULTI SCIENCES, Hangzhou, China). Briefly, cells were seeded in 6-well plates (3–4 × 10^5^/well). Transfection was carried out as described previously. After incubation with sorafenib for 48–72 h, cells were harvest and resuspended with 500 µl 1× binding buffer. After adding 5 µl annexin V-FITC and 10 µl PI, cells were incubated at room temperature in the dark for 15 min. The samples were analyzed with BD LSRFortessa cell analyzer (BD Biosciences, USA). The data analysis was performed using flowJo software.

Cell apoptosis was also determined with TUNEL assay using Direct TUNEL Apoptosis Assay Kit (MULTI SCIENCES, Hangzhou, China) according to manufacturer’s instruction. Briefly, after treatment, cells were washed twice by PBS, and fixed by 1% Polymethanol on ice for 1 h. Then cells were washed by PBS again and fixed by cold 70% ethanol in −20 °C overnight. The next day, cells were incubated with DNA binding buffer for 1 h, and PI/Rnase A Staining Buffer for another 30 min. Test was carried out by Fluorescence microscope.

### Western blot analysis

Total proteins were extracted using RIPA lysis buffer (Beyotime, Shanghai, China) supplied with protease inhibitor cocktail (MCE, USA) and phosphatase inhibitor cocktail (MCE, USA). Proteins were separated by sodium dodecyl sulfate-polyacrylamide gel electrophoresis and transferred to the PVDF membrane (Millipore, USA). Then, the membrane was blocked in skim milk (BD, USA) for 1 h at room temperature, followed by overnight at 4 °C incubating with appropriate antibody. Next day, after adequate washing in TBST and 1-h incubating with appropriate HPR-conjugated second antibody (Beyotime, Shanghai, China), the antigen-antibody complex on the membrane was detected with enhanced chemiluminescence regents (Fdbio science, Hangzhou, China). All antibodies used in this study are listed in supplementary data (Supplementary Table 1).

### qRT-PCR analysis

Total RNAs from cell or tissue samples were extracted using TRIzol (Invitrogen, USA) according to the manufacturer’s instructions. Complementary DNA (cDNA) was synthesized from 1 µg of RNA using Hifair® II 1st Strand cDNA Synthesis SuperMix for qPCR (Yeasen, Shanghai, China) or All-in-OneTM miRNA qRT-PCR (quantitative real-time PCR) Detection Kit (GeneCopoeia, USA) if the product was used for microRNA detection. qRT-PCR was performed using Hieff UNICON® qPCR SYBR Green Master Mix (Yeasen, Shanghai, China) or All-in-OneTM miRNA qRT-PCR (quantitative real-time PCR) Detection Kit (GeneCopoeia, USA). Measurement was carried out by Roche LightCycler 480 or ABI Step One. Analysis was carried out using the ΔΔCt method. Primer sequences are listed in the supplementary data (Supplementary Table 2). Normalizers used in RT-qPCR include 5 s for microRNA quantification and beta-Actin for mRNA quantification.

### Immunohistochemistry

Tumor tissue from in vivo experiment were harvested for immunohistochemistry to examine FGFR4 and EGFR expression. Immunohistochemistry was performed according to previous report^27^. Three-μm-thick sections cut from routinely processed formalin-fixed, paraffin-embedded tissue blocks were subjected to immunohistochemistry staining with specific primary antibodies against FGFR4 and EGFR, respectively. The slides were incubated with the primary antibody at 4 °C overnight. After washing with PBS, slides were subjected with detection with GTvision immunohistochemistry kit according to manufacturer’s protocol.

### Luciferase reporter assay

The 3′ untranslated region (UTR) of both EGFR and FGFR4 containing 2 potential miR-486-3p binding site were composed by TSINGKE Biological Technology (Beijing, China). sequences were listed in the supplementary data. These sequences were cloned into psiCHECK2 (Promega, USA) between XhoI and NotI. The 3′UTR was put at the end of the Renilla luciferase gene. And other reporter gene, the firefly luciferase was used as self-control. Cells transfected with miR-486-3p mimics or control were transfected with the same amount of luciferase reporter plasmid for 48–72 h. Promega Dual-Luciferase Reporter assay system (Promega, USA) was used to measure the activity of firefly and Renilla luciferase. All mutant sites were designed at the binding sites. The original binding sequence CTGCCCC in EGFR 3′UTR and CTGCCCCA in FGFR4 3′UTR were both changed to ACATATAC. Overlap extension PCR was used to manufacture the mutations. Primer sequences are listed in the supplementary data (Supplementary Table 2). Mutant sequences were cloned into plasmid using ClonExpress II One Step Cloning Kit (Vazyme, Nanjing, China)

### Target DNA encoding miR-486-3p using CRISPR/CAS9 technology

We designed CRISPR gRNAs to target the DNA sequence encoding miR-486-3p using the online software at https://portals.broadinstitute.org/gpp/public/analysis-tools/sgrna-design. Then, we synthesized oligos containing the same overhangs after BsmBI, and cloned the target sequences into the lentiCRISPRv2 (Addgene, UK)^19^. Lentivirus was constructed using the transfer plasmid lentiCRISPRv2-sgRNA and the packaging plasmids pVSVg (Addgene, UK) and psPAX2 (Addgene, UK). Supernatant containing lentivirus was collected for 48 h. Lentiviral infection of the SK-Hep-1 cell line was carried through for 24 h. After another 24 h, 1 mg/ml puromycin was added to the medium for selection.

### In vivo HCC model

In vivo experiments were conducted using 4-week-old BALB/C nude mice. orthotopic HCC model was constructed as described previously. Briefly, mice were anesthetized by pentobarbital. Abdominal median incision was used. After liver exposure, a total of 500 million SK-Hep-1 cells were implanted into one lobe of liver. The incision was closed using a suture of 5-0 silk.

After tumor establishment, tumor tissues were harvested, cut into small pieces and implanted into the livers in recipient 4-week-old BALB/C nude mice under anesthesia. One week after surgery, those mice carrying tumors started to receive sorafenib treatment.

After two-month treatment, tumors in those mice were considered to be sorafenib resistant. Subcutaneous HCC mouse model was established according to previous study^30^. Tumor tissue of one mouse was harvested, washed in PBS buffer. Necrotic tissues were removed. Tumor tissues were cut into about 1-mm^3^ pieces. One piece was implanted subcutaneously into axilla of recipient 4-week-old BALB/C nude mice.

All animal experiments were performed under the guidelines reviewed by the Animal Ethics Committee of the Biological Resource Centre of the Agency for Science, Technology and Research at the Sir Run-Run Shaw Hospital.

### Clinical data

A total of 40 randomly selected sorafenib-treated HCC patients were collected. Tumor and adjacent tumor tissues were both collected. The study conformed to the principles of the Declaration of Helsinki and was approved by the Institutional Review Board of the Sir Run-Run Shaw Hospital.

### Statistical analysis

Statistical analysis was performed using GraphPad Prism 7. Data were expressed as the mean ± standard error of the mean (SEM) from at least three independent experiments. Quantitative data between groups were compared using *t* test. OS and RFS curves were obtained by the Kaplan-Meier method, and differences were compared by log-rank test. A two-tailed *P* value of <0.05 was considered statistically significant where *p **<** 0.05; **p **<** 0.01; ***p **<** 0.001; ****p **<** 0.0001.

## Supplementary information

supplemental legend

supplemental figure1

supplemental figure2
