# Supplementary material for: miR-486-3p mediates hepatocellular carcinoma sorafenib resistance by targeting FGFR4 and EGFR
Source: Cell Death Dis. 2020 Apr 20;11(4):250. doi: 10.1038/s41419-020-2413-4 (PMC7170966; doi:10.1038/s41419-020-2413-4)
Supplement: Supplementary file 1 — supplemental legend [file 41419_2020_2413_MOESM1_ESM.docx]

**Supplementary data**

**Figure1:** clustered heatmap of significantly differentially expressed mRNAs in Huh7SR with or without miR-486-3p mimics transfection (log2 fold change >1, adjusted p <0.05). Red represented low expression, while blue represented high expression.

**Figure2**: Bubble chart of altered signaling pathways. Proteins related with FGFR4 or EGFR were determined by online database STRING or referring to literature. The size and color of each bubble represent number of differentially expressed genes in each pathway and p value respectively.

**Table1:**

Antibody:

| FGFR4 | Abcam | Ab44971 |
| --- | --- | --- |
| FGFR4 | Cell Signaling Technology | 8562 |
| EGFR | Abcam | Ab52894 |
| EGFR | Cell Signaling Technology | 2646 |
| p-ERK | Cell Signaling Technology | 4370 |
| ERK | Cell Signaling Technology | 4695 |
| Tubulin | FUDE Biological Technology | FD0064 |

**Table2:**

Primer：

| hsa-miR-671-3p | ATATTCCGGTTCTCAGGGCTC | |
| --- | --- | --- |
| hsa-miR-486-3p | CGGGGCAGCTCAGTACAG | |
| hsa-miR-486-5p | TCCTGTACTGAGCTGCCCCGAG | |
| hsa-miR-378a-3p | ACTGGACTTGGAGTCAGAAGGC | |
| hsa-miR-378a-5p | CTCCTGACTCCAGGTCCTGT | |
| hsa-miR-328-3p | CTGGCCCTCTCTGCCCTT | |
| FGFR4 | CGGGTACATTCCTCGCTCC | GACTTCCCACCAACTGCCTT |
| EGFR | TATTGATCGGGAGAGCCGGA | TCGTGCCTTGGCAAACTTTC |
| PDGFRA | AATTCCGTGGTGTTGTCG | AAGGTCCGCTGGATTGAG |
| miR-486 sgRNA | CACCGGCCTGGTTAGTGCTGCACTG | AAACCAGTGCAGCACTAACCAGGCC |
| Round1 PCR for FGFR4 mutant1-F | AATTCTAGGCGATCGCTCGAGG  CAAGGCTCAAGGCTGTGC | GACGCGTATATGTCAGCAGCAGGGGG  AGGTGTG |
| Round1 PCR for FGFR4 mutant1-R | TGCTGACATATACGCGTCTTGACG  GGAGCATTG | ATTTTATTGCGGCCAGCGGCCGCACA  CACTGGCTCCTGGGGC |
| Round2 PCR for FGFR4 mutant1 | AATTCTAGGCGATCGCTCGAGG  CAAGGCTCAAGGCTGTGC | ATTTTATTGCGGCCAGCGGCCGCACA  CACTGGCTCCTGGGGC |
| Round1 PCR for FGFR4 mutant2-F | AATTCTAGGCGATCGCTCGAGGC  AAGGCTCA | GTATATGTTGGAGGTGGCGGAGCCCA  TGG |
| Round1 PCR for FGFR4 mutant2-R | CTCCGCCACCTCCAACATATACGG  AGCCAGTGTGTGC | CGAAAAGGTCACACTCTGGGGCGC |
| Round2 PCR for FGFR4 mutant2 | AATTCTAGGCGATCGCTCGAGGC  AAGGCTCA | AAGATATTTTATTGCGGCCAGCGGCC  GCAC |
| Round1 PCR for EGFR mutant1-F | AATTCTAGGCGATCGCTCGAGCCC  ACGGTACTTACTCCCC | GCAAGACAGTATATGTCATACTGAGT  TTCAAA |
| Round1 PCR for EGFR mutant1-R | CAGTATGACATATACTGTCTTGCT  GTCATGA | TTTATTGCGGCCAGCGGCCGCTCATAAT  GCTACTGTCATTCGCAC |
| Round2 PCR for EGFR mutant1 | AATTCTAGGCGATCGCTCGAGCCC  ACGGTACTTACTCCCC | TTTATTGCGGCCAGCGGCCGCTCATAAT  GCTACTGTCATTCGCAC |
| Round1 PCR for EGFR mutant2-F | AATTCTAGGCGATCGCTCGAGCCC  ACGGTACTTACTCCCC | GGTTTGGTATATGTCTGACCTGGAGG |
| Round1 PCR for EGFR mutant2-R | GGTCAGACATATACCAAACCCCCTC | TTTATTGCGGCCAGCGGCCGCTCATAAT  GCTACTGTCATTCGCAC |
| Round2 PCR for EGFR mutant2 | AATTCTAGGCGATCGCTCGAGCCC  ACGGTACTTACTCCCC | TTTATTGCGGCCAGCGGCCGCTCATAAT  GCTACTGTCATTCGCAC |

EGFR 3’UTR: 600bp

CCCACGGTACTTACTCCCCACTGATGGACCAGTGGTTTCCAGTCATGAGCGTTAGACTGACTTGTTTGTCTTCCATTCCATTGTTTTGAAACTCAGTATGCTGCCCCTGTCTTGCTGTCATGAAATCAGCAAGAGAGGATGACACATCAAATAATAACTCGGATTCCAGCCCACATTGGATTCATCAGCATTTGGACCAATAGCCCACAGCTGAGAATGTGGAATACCTAAGGATAGCACCGCTTTTGTTCTCGCAAAAACGTATCTCCTAATTTGAGGCTCAGATGAAATGCATCAGGTCCTTTGGGGCATAGATCAGAAGACTACAAAAATGAAGCTGCTCTGAAATCTCCTTTAGCCATCACCCCAACCCCCCAAAATTAGTTTGTGTTACTTATGGAAGATAGTTTTCTCCTTTTACTTCACTTCAAAAGCTTTTTACTCAAAGAGTATATGTTCCCTCCAGGTCAGCTGCCCCCAAACCCCCTCCTTACGCTTTGTCACACAAAAAGTGTCTCTGCCTTGAGTCATCTATTCAAGCACTTACAGCTCTGGCCACAACAGGGCATTTTACAGGTGCGAATGACAGTAGCATTATGA

FGFR4 3’UTR: 600bp

GCAAGGCTCAAGGCTGTGCAGGCACATAGGCTGGTGGCCTTGGGCCTTGGGGCTCAGCCACAGCCTGACACAGTGCTCGACCTTGATAGCATGGGGCCCCTGGCCCAGAGTTGCTGTGCCGTGTCCAAGGGCCGTGCCCTTGCCCTTGGAGCTGCCGTGCCTGTGTCCTGATGGCCCAAATGTCAGGGTTCTGCTCGGCTTCTTGGACCTTGGCGCTTAGTCCCCATCCCGGGTTTGGCTGAGCCTGGCTGGAGAGCTGCTATGCTAAACCTCCTGCCTCCCAATACCAGCAGGAGGTTCTGGGCCTCTGAACCCCCTTTCCCCACACCTCCCCCTGCTGCTGCTGCCCCAGCGTCTTGACGGGAGCATTGGCCCCTGAGCCCAGAGAAGCTGGAAGCCTGCCGAAAACAGGAGCAAATGGCGTTTTATAAATTATTTTTTTGAAATAAAGCTCTGTGTGCCTGGGTCTTCCCTGAGCAACATGGAGTGGGGTGAGGTGGAGGGATCCCTCCAGCAGAGTTCTGCCTACAGGACACGGACTGAGGGCACTGGACCAGGCCATGGGCTCCGCCACCTCCACTGCCCCAGGAGCCAGTGTGT
