# Supplementary figures and images for: miR-486-3p mediates hepatocellular carcinoma sorafenib resistance by targeting FGFR4 and EGFR
Source: Cell Death Dis. 2020 Apr 20;11(4):250. doi: 10.1038/s41419-020-2413-4 (PMC7170966; doi:10.1038/s41419-020-2413-4)

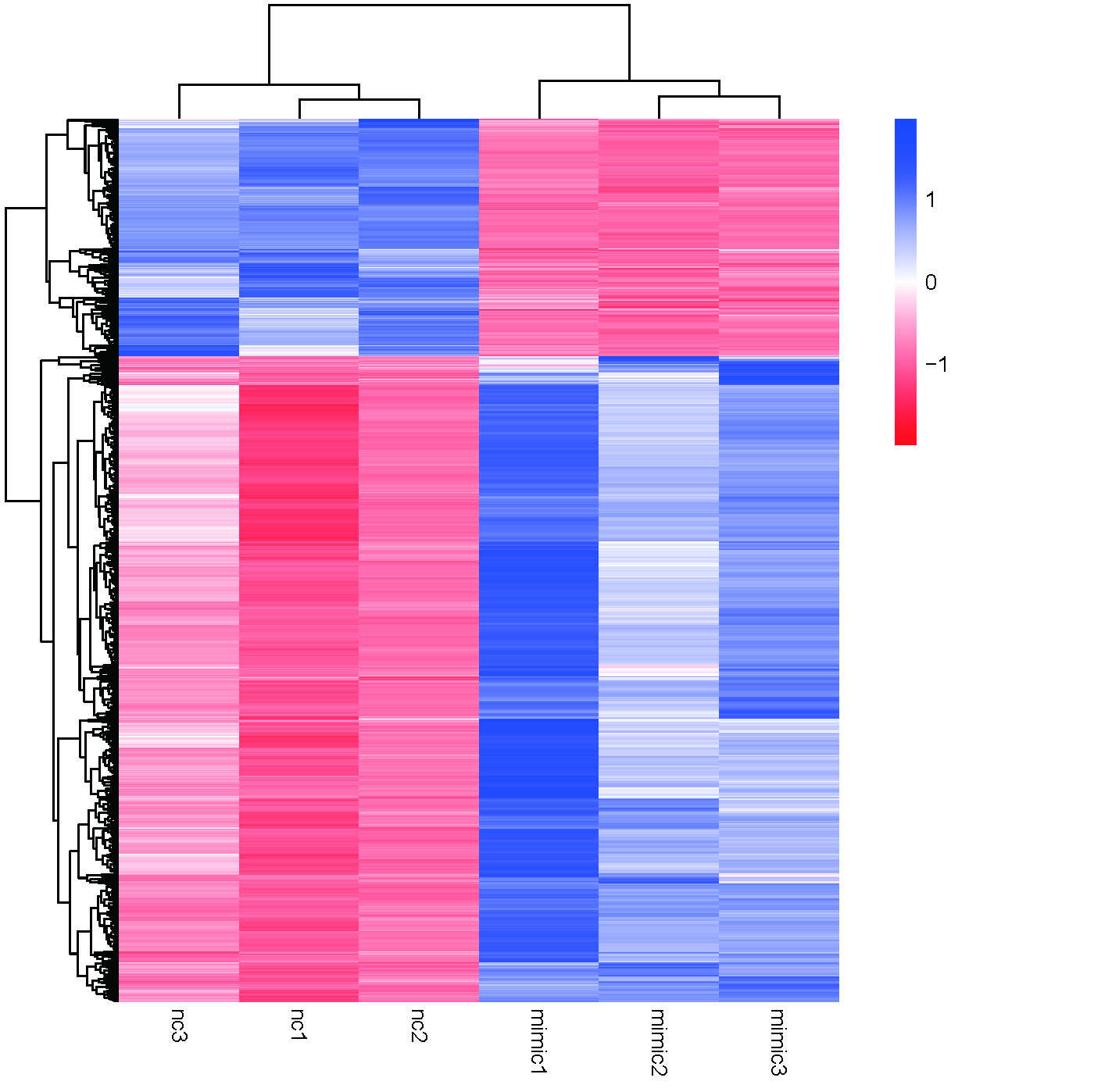

Supplement: Supplementary file 2 — supplemental figure1 [file 41419_2020_2413_MOESM2_ESM.tif]

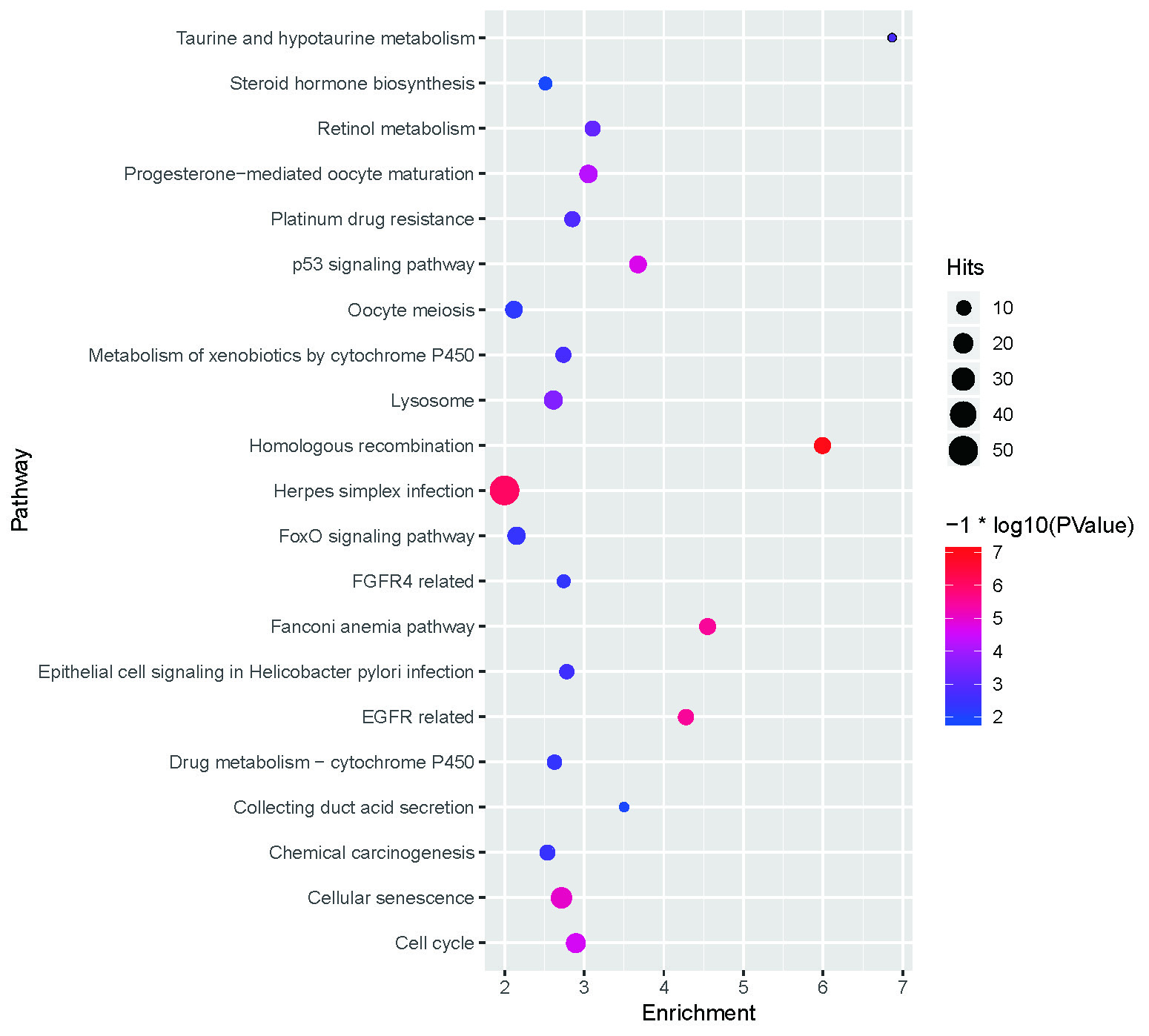

Supplement: Supplementary file 3 — supplemental figure2 [file 41419_2020_2413_MOESM3_ESM.tif]
